# Assessment of withdrawal symptoms among e-cigarette users in Saudi Arabia

**DOI:** 10.18332/tid/224315

**Published:** 2026-07-31

**Authors:** Fahad H. Alahmadi, Ahmed A. Alzahrani, Noof Aloufi, Eman Sobh, Ziyad Alshehri, Abdulrahman M. Hawsawi, Ali M. Alasmari, Keir E. J. Philip, Foad Bukhari

**Affiliations:** 1 Respiratory Therapy Department, College of Medical Rehabilitation Sciences, Taibah University, Madinah, Saudi Arabia; 2 National Heart and Lung Institute, Imperial College, London, United Kingdom; 3 Department of Clinical Laboratory Sciences, College of Applied Medical Sciences, Taibah University, Madinah, Saudi Arabia; 4 Chest Diseases Department, Faculty of Medicine for Girls, Al-Azhar University, Cairo, Egypt; 5 Department of Medicine, College of Medicine, Taibah University, Madinah, Saudi Arabia

**Keywords:** electronic cigarette, vaping, smoking, withdrawal symptoms

## Abstract

**INTRODUCTION:**

E-cigarette use or ‘vaping’ is increasing globally. Withdrawal symptoms have been extensively researched in tobacco users, but less is known regarding withdrawal among e-cigarette users. We aimed to explore the type, relative frequency, and severity of withdrawal symptoms experienced by e-cigarette users in Saudi Arabia.

**METHODS:**

A cross-sectional survey was administered to e-cigarette users aged ≥18 years residing in Saudi Arabia in 2023, using a convenience sampling frame. The primary outcome is the withdrawal symptoms. Descriptive statistics and correlations were employed to analyze the data.

**RESULTS:**

Data from 339 participants were analyzed: 87% males, 66% students, 90% used nicotine-containing e-cigarettes, with 55% using higher strength nicotine concentrations (>18 mg/mL). The most frequently reported psychological withdrawal symptoms were intense cravings for nicotine (43%), anger (29%), restlessness (28%), and poor concentration (20%), while 71% of participants reported experiencing at least one withdrawal symptom. The most commonly reported physical withdrawal symptoms were headache (34%), dizziness (17%), heart palpitations (15%), and breathing difficulties (14%). Approximately 42% of participants reported moderate-to-severe withdrawal symptoms. Notably, 36.9% returned to smoking to alleviate withdrawal symptoms.

**CONCLUSIONS:**

E-cigarette users in Saudi Arabia experienced a range of psychological and physical withdrawal symptoms. Psychological symptoms were more commonly reported than physical ones. Further studies are warranted to elucidate the factors influencing the occurrence and severity of these symptoms.

## INTRODUCTION

E-cigarette use or vaping has gained popularity worldwide, especially among young adults^1^. E-cigarettes are the most reported type of tobacco product use among school students in the national tobacco survey in the US^2^. Similarly, in Saudi Arabia, several studies have reported high levels of e-cigarette use among young adults^3-5^. Emerging evidence has suggested that e-cigarettes can lead to nicotine addiction and pose various health risks^2,6,7^. One crucial aspect of e-cigarette addiction is the experience of withdrawal symptoms upon cessation or reduction of use^8^. Withdrawal symptoms from nicotine, the primary addictive substance in e-cigarettes, can manifest both physically and psychologically. Common physical symptoms include weight gain, sweating, abdominal cramping, tremor, breathing difficulty, constipation, sore or itchy throat, heart palpitations, dizziness, headache, and nausea. Psychological symptoms refer to social isolation, depression, feeling sleepy, poor concentration, anger, stress, restlessness, and intense cravings^8,9^. Understanding the prevalence, type, and severity of withdrawal symptoms among e-cigarette users is essential for developing effective cessation strategies and public health interventions. Previous studies primarily focused on withdrawal symptoms among traditional tobacco cigarette smokers. However, the expanding prevalence of e-cigarette use necessitates a deeper understanding of withdrawal experiences specific to this population. Furthermore, the experience of withdrawal may have cultural differences; therefore, research from a range of different countries is required. Currently, research on the prevalence of e-cigarette withdrawal symptoms is limited^8-11^. To our best knowledge, no previous studies have been conducted in Saudi Arabia. This study aims to address this gap by investigating the prevalence, type, and severity of withdrawal symptoms among e-cigarette users in Saudi Arabia.

## METHODS

### Study design and participants

This is a descriptive cross-sectional study. The inclusion criteria for participants included being a resident of Saudi Arabia, aged ≥18 years, and being an e-cigarette user. Those who smoke conventional cigarettes in addition to e-cigarettes were excluded from the study to avoid confusing e-cigarette withdrawal symptoms with those from traditional tobacco cigarettes. According to the latest census in 2021, there are about 32 million people living in Saudi Arabia^12^. The sample size was calculated using an anticipated prevalence (proportion) of 26.3% for withdrawal symptoms of e-cigarettes based on a previous study^5^ . We used an electronic sample size calculator^13^ with a 5% margin of error and a 95% confidence level, which yielded a minimum sample size of 298. To compensate for possible incomplete or poor-quality data, we increased the sample by 5%, giving a final target of 311 participants. A non-random convenience sampling method was employed.

### Study tool and data collection

The study tool is a validated and reliable Arabic version of a questionnaire developed by Barakat et al.^9^, with permission from the authors. The survey was administered to participants via an online Google Form from March to October 2023, through social media platforms (we distributed the questionnaire through general groups and asked them to share it on e-cigarette users’ platforms).

The survey is composed of three parts. The demographic data section included age, gender, education level, country of residence, and smoking status. The e-cigarette use information section contained seven questions related to e-cigarette use, such as reasons for starting, nicotine concentration and volume, and the type of e-cigarette device. The e-cigarette withdrawal symptoms section included a list of withdrawal symptoms (eight psychological symptoms and 11 physical symptoms), the severity and duration of withdrawal symptoms, the onset time of withdrawal symptoms after the last e-cigarette use, and measures used to alleviate the symptoms. The respondent could choose more than one symptom.

### Statistical analysis

We imported data from Google Forms into Microsoft Excel (Microsoft Office 365, Microsoft Corporation, US). We used the Statistical Package for the Social Sciences (IBM SPSS Statistics for Macintosh, Version 29.0.2.0, Armonk, NY: IBM Corp) to analyze the data in the study. For descriptive analyses, data are reported as frequencies and percentages. We used Spearman’s correlation to assess the relationship between withdrawal symptoms and the volume, type, and concentration of nicotine in e-cigarette liquid per day. A p<0.05 was considered statistically significant.

## RESULTS

**Figure 1:**
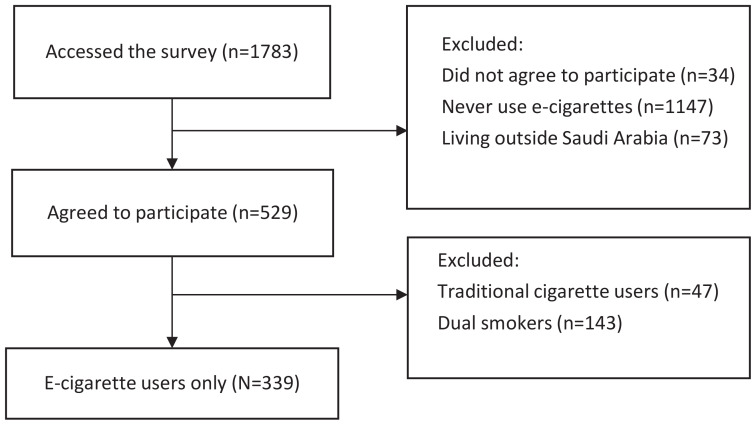
Flowchart of the study, a cross-sectional survey, Saudi Arabia 2023 (N=339)

We received 1750 responses. Of them, 339 met the study inclusion and exclusion criteria (Figure 1).

Most participants were male (87%), aged 18–25 years (79%), and students (65%). As per the inclusion criteria, all participants were e-cigarette users. Few (9.6%) used nicotine-free e-cigarette liquid, while most of them (90%) used e-cigarettes containing either salted (49.6%) or unsalted (40.9%) nicotine, with 55% of the participants using higher nicotine concentrations (>18 mg/mL) (Table 1).

**Table 1:** Sociodemographic characteristics of the study participants, a cross-sectional survey, Saudi Arabia 2023 (N=339)

*Characteristics*	*n (%)*
**Age** (years)	
18–25	269 (79.4)
26–40	57 (16.8)
>40	13 (3.8)
**Sex**	
Male	296 (87.3)
Female	43 (12.7)
**Education level**	
Primary	3 (0.9)
Intermediate	10 (2.9)
High/Diploma	151 (44.5)
Bachelor’s degree	160 (47.2)
Postgraduate	15 (4.4)
**Occupation**	
Employed	86 (25.4)
Student	220 (64.9)
Unemployed	33 (9.7)
**E-liquid type**	
Nicotine free	32 (9.6)
Unsalted nicotine	137 (40.9)
Salted nicotine	166 (49.6)
**Concentration of nicotine** (mg/mL)	
≤6	95 (28.4)
>6 to18	23 (6.9)
>8 to 30	67 (20)
>30 to 40	11 (3.3)
>40	107 (31.9)
**Consumed e-liquid per day** (mL)	
<2	175 (51.6)
2–5	129 (38.1)
>5	35 (10.3)

### Withdrawal symptoms

**Figure 2:**
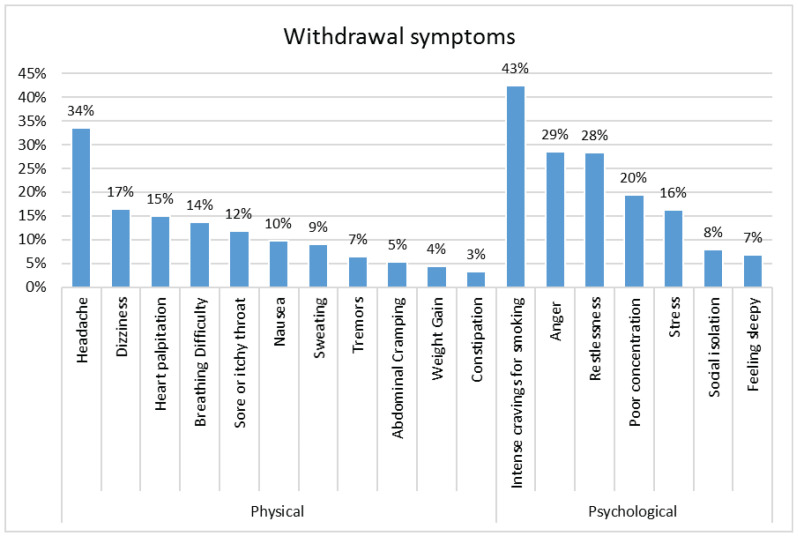
Withdrawal symptoms among the study population, a cross-sectional survey, Saudi Arabia 2023 (N=339)

About 55% of participants reported withdrawal symptoms, and 25% were not sure if they had withdrawal symptoms. However, 71% reported they had at least one withdrawal symptom. Psychological symptoms were more common (61.4%) than physical symptoms (56.6%). About 42% of participants reported moderate-severe/very severe withdrawal symptoms, and 36.9 % returned to smoking as a measure to alleviate withdrawal symptoms (Table 2). Intense craving for smoking was the most common psychological symptom (43%), and headache was the most common physical symptom (34%) (Figure 2).

**Table 2:** Withdrawal symptoms type, severity, onset, and measures to overcome, a cross-sectional survey, Saudi Arabia 2023 (N=339)

*Withdrawal symptoms*	*n (%)*
**Type of symptoms**	
At least one symptom	241 (71.1)
Physical symptoms	192 (56.6)
Psychological symptoms	208 (61.4)
**The severity of the withdrawal symptoms from the participants’ perception** (N=203)	
Weak, mild	117 (58.0)
Moderate, severe, very severe	86 (42.0)
**Starting time of withdrawal symptoms from the last e-cigarette use** (hours) (N=194)	
<6	44 (22.7)
6–16	75 (38.6)
>16	75 (38.7)
**Duration of symptoms before returning to smoking** (days) (N=190)	
1–2	112 (59.0)
3–7	59 (31.0)
>7	19 (10.0)
**Measures to alleviate withdrawal symptoms** (N=225)	
Taking medications to manage the symptoms	21 (9.3)
Using nicotine replacement therapy (patch)	15 (6.7)
Go back to smoking	83 (36.9)
Keeping myself busy to tolerate those withdrawal symptoms	92 (40.9)
Taking advice (counseling) from a pharmacist or medical health staff	11 (4.9)
Other	3 (1.3)

The increased number of withdrawal symptoms was significantly associated with increased volume of nicotine consumed per day (r=0.118, p=0.029). The presence of three or more psychological withdrawal symptoms was correlated with increased volume of nicotine consumed per day (r=0.18, p<0.001), nicotine salt (r=0.12, p=0.028), and concentration of nicotine in the liquid consumed (r=0.107, p=0.049). The severity of withdrawal symptoms was correlated with increased volume of nicotine consumed per day (r=0.209, p<0.001), nicotine salt use (r=0.144, p=0.008), and concentration of nicotine in the liquid consumed (r=0.137, p=0.012).

## DISCUSSION

The findings of this study reveal the prevalence, type, and severity of withdrawal symptoms experienced by e-cigarette users in Saudi Arabia. Most participants reported experiencing at least one withdrawal symptom, which further highlights the addictive nature of e-cigarettes. The high prevalence of withdrawal symptoms observed in this study is consistent with previous studies^8,9^. This suggests that e-cigarettes, despite being perceived as a less harmful alternative to cigarettes, can lead to significant dependence and withdrawal symptoms.

Our results are broadly consistent with previous related research. Hughes et al.^8^ conducted an un-blinded clinical trial investigating withdrawal symptoms in e-cigarette users, and found significant withdrawal symptoms related to abstinence. Barakat et al.^9^ found that more than half of the participants in their study experienced withdrawal symptoms from e-cigarettes. The present study also showed that psychological withdrawal effects were more prevalent than physical symptoms (61.4% and 56.6%, respectively). Craving for smoking was the most prevalent psychological symptoms (43%), and headache was the most common physical symptom (34%). These results are in line with those of previous studies, such as the withdrawal symptoms documented in the study of Hughes et al.^8^. Barakat et al.^9^ found that craving was also common and that over half of the participants had withdrawal symptoms during intermittent fasting while headache was less common. Additionally, Rostron et al.^11^ found that more than one-third of the exclusive e-cigarette users reported severe craving symptoms. Exclusive e-cigarette users were less likely to experience withdrawal symptoms and cravings (46.2%) when compared to conventional cigarette smokers (61.8%), while dual conventional and e-cigarette users experienced an increase in cravings (77.1%)^11^.

Our results suggest that addiction to e-cigarettes may be comparable in severity to that of tobacco cigarettes. This finding is consistent with prior research, which has shown that psychological dependence plays a significant role in e-cigarette addiction, and craving is one of the most important signs for failure of smoking cessation trials^14^.

In the current study, most participants experienced more than one withdrawal symptom (71.1%), and the severity of symptoms was weak to mild in 58% of participants. Symptoms appeared early in less than 16 hours (61.3%). About 60% of participants returned to smoking within two days from the onset of withdrawal symptoms, while about 30% returned to smoking within seven days from the onset of the symptoms. These results are in line with previous studies, which reported that nicotine withdrawal symptoms usually reach the maximum intensity in the first 48 hours of stopping smoking.

Nearly 50% of participants experienced more than three symptoms, and a low percentage (10%) of participants reported strong craving symptoms^8^. In our study, the severity of withdrawal symptoms reported by most individuals (58%) was weak to mild. Similar results were reported by Barakat et al.^9^. The substantial number of participants who reported moderate-to-very severe withdrawal symptoms in this study (42%) emphasizes the necessity of successful and effective cessation strategies to assist people in overcoming their dependence on e-cigarettes. The high prevalence of psychological symptoms, particularly craving, among the current study participants may be attributed to the high nicotine content and dose. Nicotine is well-known to be addictive; flavoring additives can increase the addictive impact by minimizing the taste of nicotine, making it more tempting. E-cigarettes result in fast and high levels of nicotine, which may lead to dependence^8^. The relationship found in this study between the number and severity of withdrawal symptoms and the volume of nicotine consumed per day may reflect nicotine dependence. Recent studies reported higher nicotine dependence of e-cigarette users compared to traditional cigarette users^15^. A recent US tobacco survey reported increased intensity of e-cigarettes as well as nicotine dependence over time from 2017 to 2021 for sole e-cigarette users^16^.

Rapid onset of effects and the dose are considered the main predictors of physical dependence on nicotine^17^ and e-cigarettes fulfil these criteria if sufficient concentration and dose of nicotine are used. Furthermore, some e-cigarette users produce nicotine levels comparable to traditional cigarette smokers and have high nicotine uptake^18^. These findings may indicate that these dual users are more tobacco-dependent than exclusive cigarette smokers. This result would be consistent with data from observational studies suggesting that cigarette smokers who have used e-cigarettes are less likely to quit smoking than smokers who have not used e-cigarettes^19^.

In this study, all participants who had withdrawal symptoms reported onset within the first 24 hours following e-cigarette cessation, and most of them indicated that symptoms lasted for 1–2 days before returning to smoking (59%), indicating intense craving. One-third of participants’ symptoms lasted several days, and 10% of them had withdrawal symptoms that lasted more than one week. Nicotine craving is reported to start within minutes to hours and may last for several weeks^20,21^. The high prevalence of withdrawal symptoms may have an impact on everyday functioning, which emphasizes the significance of addressing e-cigarette addiction. Withdrawal symptoms can drastically reduce quality of life and may lead to relapse. Future research should explore factors associated with and influencing the severity of withdrawal symptoms. Additionally, studies investigating the effectiveness of different cessation methods for e-cigarette users are warranted.

### Strengths and limitations

This study has some key strengths. To our knowledge, this is the first study to explore e-cigarette withdrawal symptoms in Saudi Arabia, which is an increasingly important public health issue. Secondly, using a validated questionnaire improves confidence in our findings and our ability to compare our results with others using the same methodology. Thirdly, participants were exclusive e-cigarette users, improving our confidence that symptoms were related to e-cigarettes rather than other products.

Certain limitations should be discussed. Firstly, the cross-sectional observational design limits the ability to draw causal inferences. Longitudinal studies would be useful to investigate the long-term effects of e-cigarette use and withdrawal. Our analyses were descriptive and presented without stratification into subgroups (e.g. presence vs absence of withdrawal symptoms). The study was not designed or powered to perform subgroup comparisons, which may limit the depth of interpretation.

Future studies with larger samples could explore such stratified analyses to provide more details. Secondly, the reliance on self-reported data may introduce bias. Despite attempting to adjust for factors including type, dose, and concentration of nicotine, these were self-reported, which may carry recall and social desirability bias. Future studies could incorporate objective measures, such as biological markers, to assess withdrawal symptoms. Additionally, given our data collection methods and sample size, it is difficult to be sure that this is a truly representative sample, given potentially relevant factors including social media use and digital literacy, which limit the generalizability of results. However, the relatively low age and male predominance of participants broadly reflect the limited data that exist on e-cigarette and traditional cigarette use in the region more generally.

## CONCLUSIONS

This study shows that e-cigarette abstinence among e-cigarette users is associated with frequent withdrawal symptoms. Psychological withdrawal symptoms were more prevalent than physical symptoms. Craving and headaches were the most common symptoms. Though the severity of symptoms was weak to mild in most participants, a high percentage returned to smoking within one week of abstinence. Despite the limitations, this study provides a better understanding of e-cigarette addiction in Saudi Arabia.

## Data Availability

The data supporting this research are available from the authors on reasonable request.
